# Data and performance of an active-set truncated Newton method with non-monotone line search for bound-constrained optimization

**DOI:** 10.1016/j.dib.2018.11.061

**Published:** 2018-11-20

**Authors:** A. Cristofari, M. De Santis, S. Lucidi, F. Rinaldi

**Affiliations:** a Dipartimento di Matematica, Università di Padova, Via Trieste, 63, 35121 Padua, Italy; b Dipartimento di Ingegneria Informatica, Automatica e Gestionale Sapienza Università di Roma, Via Ariosto, 25, 00185 Rome, Italy

## Abstract

In this data article, we report data and experiments related to the research article entitled “A Two-Stage Active-Set Algorithm for Bound-Constrained Optimization”, by Cristofari et al. (2017). The method proposed in Cristofari et al. (2017), tackles optimization problems with bound constraints by properly combining an active-set estimate with a truncated Newton strategy. Here, we report the detailed numerical experience performed over a commonly used test set, namely CUTEst (Gould et al., 2015). First, the algorithm ASA-BCP  proposed in Cristofari et al. (2017) is compared with the related method NMBC (De Santis et al., 2012). Then, a comparison with the renowned methods ALGENCAN (Birgin and Martínez et al., 2002) and LANCELOT B (Gould et al., 2003) is reported.

## Specifications table

**Table:** 

Subject Area	Operations Research and Management Science
More specific subject area	Nonlinear Optimization
Type of data	Tables
How data was acquired	http://www.cuter.rl.ac.uk/, experimental output data
Data format	Raw and filterd
Experimental factors	None
Experimental features	A comparison of different solvers for bound-constrained optimization problems on CUTEst test set is reported.
Data accessibility	Test problems are available at http://www.cuter.rl.ac.uk/. Further details on output data available on https://sites.google.com/a/dis.uniroma1.it/asa-bcp/ and at request to the authors.

## Value of the data

• Output data reported represent a benchmark for future comparisons, among algorithms for box constrained optimization.
• Output data can be used by other researchers for tuning parameters related to active-set strategies and truncated Newton methods.
• Output data highlights how non-monotone line search procedures can be used in combination with active-set strategies.

## 1 Data

We report the data related to the numerical experience carried out to assess the performance of algorithm ASA-BCP  proposed in [3]. All computations have been run on an Intel Xeon(R), CPU E5-1650 v2 3.50 GHz. The test set consists of 140 bound-constrained problems from the CUTEst collection [10], with dimension up to $10^{5}$. The stopping condition for all codes is

$$\|x-{\left[x-g(x)\right]}^{♯}{\|}_{\infty }<10^{-5},$$

where g(·) and $\|\cdot {\|}_{\infty }$ denote the gradient of the objective function, and the sup-norm of a vector, respectively. In the following, we further denote by ${\left[\cdot \right]}^{♯}$ the projection of a vector onto the feasible set $[l,u]\subset {\mathrm{IR}}^{n}$.

Following the analysis suggested in [1], we checked whether the codes find different stationary points. The results are then reported distinguishing if all codes find the same stationary point (with a tolerance of $10^{-3}$) or not.

## 2 Experimental design, materials and methods

In the following, we provide the implementation details related to ASA-BCP, according to the algorithmic scheme reported in [3]: ASA-BCP  is a two-stage algorithmic framework that suitably combines the active-set estimate proposed in [7] with the non-monotone line search procedure described in [5] to handle box constrained optimization problems.

In the first stage of ASA-BCP, an active-set estimate is employed to detect those variables that are at the bounds at a stationary point. The active-set estimate has been used also in the context of mixed-integer convex quadratic programming [2] and of $\ell_{1}$-regularized problems [6] and depends on a parameter ϵ. In Section 2.1, we detail how to properly update ϵ in order to satisfy the assumptions of Proposition 3.5 in [3].

In the second stage of ASA-BCP, a truncated Newton strategy is used in the subspace of the variables estimated non-active. Details on how we compute the search direction for the minimization with respect to the estimated non-active variables are reported in Section 2.2.

The following notation will be used. Given a feasible point $x^{k}$ produced by ASA-BCP, we write

$$A_{l}^{k}\;≔\;A_{l}(x^{k}),\;A_{u}^{k}\;≔\;A_{u}(x^{k})\;\text{and}\;N^{k}\;≔\;N(x^{k})$$

to denote the set of estimated active and non-active sets at $x^{k}$. In particular, $A_{l}^{k}$ will denote the set of variable estimated to be active at lower bound l and $A_{u}^{k}$ will denote the set of variable estimated to be active at lower bound u. Similarly, given a feasible point ${\overset{˜}{x}}^{k}$ produced by ASA-BCP, we write

$${\overset{˜}{A}}_{l}^{k}\;≔\;A_{l}({\overset{˜}{x}}^{k}),\;{\overset{˜}{A}}_{u}^{k}\;≔\;A_{u}({\overset{˜}{x}}^{k})\;\text{and}\;{\overset{˜}{N}}^{k}\;≔\;N({\overset{˜}{x}}^{k}).$$

### 2.1 Updating of the ϵ parameter in ASA-BCP

A feature that characterizes ASA-BCP  is the use of the active-set estimate: once we have an ϵ satisfying Assumption 3.1 in [3], a decrease of the objective function is guaranteed by fixing the estimated active variables to the values at the bounds (as it is shown in Proposition 3.5 in [3]).

In general, such an ϵ cannot be “a priori” computed. This is why in our implementation we use the following updating rule. Starting from the value $\epsilon \;≔\;\min\{10^{-6},\|x^{0}-{\left[x^{0}-g(x^{0})\right]}^{♯}{\|}^{-3}\}$, at every iteration k we compute $A_{l}^{k}$, $A_{u}^{k}$ and $N^{k}$. Then, we get the point

$${\overset{˜}{x}}_{A_{l}^{k}}^{k}\;≔\;l_{A_{l}^{k}},\;{\overset{˜}{x}}_{A_{u}^{k}}^{k}\;≔\;u_{A_{u}^{k}},\;{\overset{˜}{x}}_{N^{k}}^{k}\;≔\;x_{N^{k}}^{k}.$$

If ${\overset{˜}{x}}^{k}$ satisfies

$$f({\overset{˜}{x}}^{k})-f(x^{k})\leq -\frac{1}{2\epsilon }\|{\overset{˜}{x}}^{k}-x^{k}{\|}^{2},$$

then we accept ${\overset{˜}{x}}^{k}$ and do not change the value of ϵ. Otherwise, we do not accept ${\overset{˜}{x}}^{k}$, we reduce ϵ and estimate the active variables again, repeating this procedure until the above relation is satisfied.

### 2.2 Calculation of the search direction in ASA-BCP

At every iteration k, in order to compute the search direction with respect to ${\overset{˜}{N}}^{k}$, ASA-BCP  approximately solves the Newton equation

$$H_{{\overset{˜}{N}}^{k}{\overset{˜}{N}}^{k}}({\overset{˜}{x}}^{k})d_{{\overset{˜}{N}}^{k}}^{k}+g_{{\overset{˜}{N}}^{k}}({\overset{˜}{x}}^{k})=0$$

by using the conjugate gradient strategy considered in [4]. In particular, let $m=|{\overset{˜}{N}}^{k}|$, the scheme produces a finite sequence of conjugate directions $p_{0},p_{1},\ldots ,p_{i}\in {\mathrm{IR}}^{m}$, i<m, and the approximated Newton direction $d_{{\overset{˜}{N}}^{k}}^{k}$ is computed as $d_{{\overset{˜}{N}}^{k}}^{k}\;≔\;d_{i+1}$, where

$$d_{i+1}≔-\overset{i}{\underset{j=0}{\sum }}\frac{g_{{\overset{˜}{N}}^{k}}{\left({\overset{˜}{x}}^{k}\right)}^{T}p_{j}}{p_{j}^{T}H_{{\overset{˜}{N}}^{k}{\overset{˜}{N}}^{k}}({\overset{˜}{x}}^{k})p_{j}}p_{j}.$$

In our implementation of ASA-BCP, we employed the following stopping criterion for the conjugate gradient method:

$$\left|\frac{\left[q(d_{i+1})-q(d_{i})]-\frac{3}{2}[g_{{\overset{˜}{N}}^{k}}{\left({\overset{˜}{x}}^{k}\right)}^{T}d_{i+1}-g_{{\overset{˜}{N}}^{k}}{\left({\overset{˜}{x}}^{k}\right)}^{T}d_{i}\right]}{q(d_{i+1})-\frac{3}{2}g_{{\overset{˜}{N}}^{k}}{\left({\overset{˜}{x}}^{k}\right)}^{T}d_{i+1}}\right|(i+1)\leq \gamma_{k},$$

where

$$q(d)\;≔\;\frac{1}{2}d^{T}H_{{\overset{˜}{N}}^{k}{\overset{˜}{N}}^{k}}({\overset{˜}{x}}^{k})d+g_{{\overset{˜}{N}}^{k}}{\left({\overset{˜}{x}}^{k}\right)}^{T}d,$$

and $\gamma_{k}$ is taken as

$$\left\{ \begin{array}{cc} \eta_{k}, & \text{if}\;\|d_{i+1}\|\geq \|g_{{\overset{˜}{N}}^{k}}({\overset{˜}{x}}^{k})\|,\\ \eta_{k}\min\{1,\|d_{i+1}\|\}, & \mathrm{otherwise}. \end{array}\right.$$

In ASA-BCP, we set $\eta_{k}=\min\left\{1,\;0.1+e^{-0.001\;k}\right\}$.

### 2.3 Algorithm parameters used in the comparisons

In the following, we report the parameters values of the algorithms we consider in [3] for the comparisons.

For all methods, the stopping condition is

$$\|x-{\left[x-g(x)\right]}^{♯}{\|}_{\infty }<10^{-5}.$$

In running ASA-BCP, according to the algorithmic scheme reported in [3], we set Z≔20 and M≔99 (so that, in the non-monotone line search procedure, the last 100 objective function values are included in the computation of the reference value).

In running the other methods, default values are used for all parameters. More specifically:

• In NMBC [5], a non-monotone strategy is employed (it is similar to the one described for ASA-BCP), where the parameters Z and M are equal to 20 and 100, respectively. Moreover, the parameter ϵ used in the active-set estimate is equal to $10^{-4}$.
• In ALGENCAN [8], the truncated Newton method is used as inner solver method, the scaling feature is disabled, and the parameter η is equal to 0.1 (a face of the feasible set is abandoned by the algorithm when the norm of the internal components of the continuous projected gradient is smaller than η times the norm of the continuous projected gradient).
• In LANCELOT B[9], a band preconditioner is employed for the conjugate gradient method, with a semi-bandwidth equal to 5. Moreover, a non-monotone strategy is used with a history-length equal to 1.

### 2.4 Comparison between ASA-BCP and NMBC

In Table 1, we report the numerical results obtained by ASA-BCP  and NMBC  on 140 problems from the CUTEst collection.

**Table 1:** Comparison between ASA-BCP  and NMBC on 140 problems from the CUTEst collection.

**Problem**	n	**ASA-BCP**				**NMBC**			
		obj	f-eval	cg-it	time (s)	obj	f-eval	cg-it	time (s)
BDEXP	1000	3.953e−04	11	10	0.0	3.940e−04	3	20	0.0
BDEXP	5000	1.967e−03	11	10	0.0	1.966e−03	3	20	0.1
BIGGSB1	1000	1.500e−02	198	2272	0.1	1.500e−02	5	1288	0.1
BIGGSB1	5000	1.595e−02	199	2442	0.6	1.500e−02	6	3974	0.9
BIGGSB1	10,000	1.595e−02	199	2442	1.3	1.500e−02	6	6598	2.9
BIGGSB1	20,000	1.595e−02	199	2442	2.5	1.500e−02	7	19,025	16.6
BQPGAUSS	2003	−3.626e−01	9	9040	1.8	−3.626e−01	60	7674	1.6
CHARDIS0	2000	6.384e−22	2	1	0.1	2.260e−19	2	2	0.1
CHARDIS0	4000	4.648e−20	2	1	0.5	4.398e−18	2	2	0.5
CHARDIS0	10,000	1.246e−19	2	1	2.8	1.526e−16	2	2	3.3
CHENHARK	1000	−2.000e+00	107	1953	0.1	−2.000e+00	107	56,216	2.2
CHENHARK	5000	−2.000e+00	73	1556	0.4	−2.000e+00	89	129,087	23.0
CHENHARK	10,000	−2.000e+00	108	1935	0.9	−2.000e+00	117	278,775	105.7
CHENHARK	50,000	−2.000e+00	137	2643	4.5	−2.000e+00	61	207,468	348.6
CVXBQP1	1000	2.252e+04	10	9	0.0	2.252e+04	4	16	0.0
CVXBQP1	10,000	2.250e+06	13	11	0.0	2.250e+06	4	19	0.0
CVXBQP1	100,000	2.250e+08	15	12	0.2	2.250e+08	4	22	0.2
EXPLIN	1200	−7.193e+07	108	279	0.0	−7.193e+07	119	279	0.0
EXPLIN2	1200	−7.200e+07	41	69	0.0	−7.200e+07	106	205	0.0
EXPQUAD	1200	−3.685e+09	146	225	0.0	−3.685e+09	106	320	0.1
JNLBRNG1	1024	−1.803e−01	4	135	0.0	−1.803e−01	3	171	0.1
JNLBRNG1	1156	−1.803e−01	3	128	0.0	−1.803e−01	3	184	0.1
JNLBRNG1	5625	−1.805e−01	4	278	0.4	−1.805e−01	8	483	0.8
JNLBRNG1	10,000	−1.806e−01	5	391	1.1	−1.806e−01	11	699	1.9
JNLBRNG1	15,625	−1.806e−01	5	429	1.6	−1.806e−01	13	955	3.7
JNLBRNG2	1024	−4.125e+00	3	158	0.0	−4.125e+00	3	167	0.1
JNLBRNG2	1156	−4.128e+00	3	191	0.1	−4.128e+00	3	146	0.1
JNLBRNG2	5625	−4.147e+00	5	407	0.5	−4.147e+00	6	441	0.7
JNLBRNG2	10,000	−4.149e+00	6	527	1.3	−4.149e+00	8	752	1.9
JNLBRNG2	15,625	−4.150e+00	7	659	2.3	−4.150e+00	10	969	3.4
JNLBRNGA	1024	−2.954e−01	3	99	0.0	−2.954e−01	3	125	0.0
JNLBRNGA	1156	−2.935e−01	3	120	0.0	−2.935e−01	3	142	0.1
JNLBRNGA	5625	−2.753e−01	5	340	0.5	−2.753e−01	8	405	0.6
JNLBRNGA	10,000	−2.711e−01	6	418	1.1	−2.711e−01	11	650	1.7
JNLBRNGA	15,625	−2.685e−01	5	446	1.8	−2.685e−01	13	907	3.4
JNLBRNGB	1024	−6.440e+00	4	596	0.1	−6.440e+00	4	449	0.1
JNLBRNGB	1156	−6.429e+00	4	593	0.1	−6.429e+00	3	524	0.1
JNLBRNGB	5625	−6.330e+00	6	1463	1.7	−6.330e+00	4	1388	1.7
JNLBRNGB	10,000	−6.301e+00	7	1962	3.7	−6.301e+00	4	2333	4.6
JNLBRNGB	15,625	−6.281e+00	8	2361	7.1	−6.281e+00	7	2902	8.9
LINVERSE	1999	6.810e+02	21	241	0.1	6.810e+02	31	422	0.2
MCCORMCK	1000	−9.137e+02	7	20	0.0	−9.137e+02	38	1019	0.2
MCCORMCK	5000	−4.567e+03	13	22	0.0	−4.567e+03	39	765	0.8
MCCORMCK	10,000	−9.133e+03	12	16	0.1	−9.133e+03	38	547	1.1
MCCORMCK	50,000	−4.566e+04	18	29	0.5	−4.566e+04	37	255	2.5
MINSURFO	2706	2.515e+00	5	274	0.3	2.515e+00	26	2390	2.6
MINSURFO	5931	2.485e+00	105	647	1.8	2.485e+00	27	1994	4.2
NCVXBQP1	1000	−1.987e+08	10	9	0.0	−1.987e+08	10	10	0.0
NCVXBQP1	10,000	−1.986e+10	11	10	0.0	−1.986e+10	11	12	0.0
NCVXBQP1	100,000	−1.985e+12	15	11	0.2	−1.985e+12	11	12	0.2
NCVXBQP2	1000	−1.334e+08	15	24	0.0	−1.334e+08	13	28	0.0
NCVXBQP2	10,000	−1.334e+10	31	63	0.1	−1.334e+10	30	70	0.1
NCVXBQP2	100,000	−1.334e+12	41	100	0.7	−1.334e+12	39	101	0.7
NCVXBQP3	1000	−6.530e+07	20	34	0.0	−6.530e+07	20	34	0.0
NCVXBQP3	10,000	−6.506e+09	54	129	0.1	−6.506e+09	6164	15,326	9.8
NCVXBQP3	100,000	−6.505e+11	287	1024	6.6	−6.505e+11	679	3076	16.7
NOBNDTOR	5476	−4.499e−01	4	235	0.4	−4.499e−01	10	492	0.8
NOBNDTOR	10,000	−4.438e−01	5	318	0.9	−4.438e−01	16	763	2.3
NOBNDTOR	14,884	−4.405e−01	6	396	1.7	−4.405e−01	19	914	3.8
NOBNDTOR	40,000	−4.347e−01	9	659	7.6	−4.347e−01	34	1981	20.9
NONSCOMP	1000	8.661e−16	7	37	0.0	1.869e−12	3	29	0.0
NONSCOMP	5000	5.054e−13	7	34	0.0	1.038e−19	3	42	0.0
NONSCOMP	10,000	1.680e−12	7	37	0.0	7.380e−19	3	41	0.0
OBSTCLAE	5625	1.863e+00	9	221	0.4	1.863e+00	3	293	0.4
OBSTCLAE	10,000	1.886e+00	23	438	1.6	1.886e+00	5	384	0.9
OBSTCLAE	15,625	1.901e+00	16	406	1.9	1.901e+00	9	541	1.8
OBSTCLAL	5625	1.863e+00	4	124	0.2	1.863e+00	3	215	0.2
OBSTCLAL	10,000	1.886e+00	4	159	0.3	1.886e+00	5	317	0.6
OBSTCLAL	15,625	1.901e+00	4	211	0.7	1.901e+00	8	433	1.2
OBSTCLBL	5625	7.231e+00	4	116	0.2	7.231e+00	5	183	0.3
OBSTCLBL	10,000	7.272e+00	4	155	0.5	7.272e+00	6	273	0.7
OBSTCLBL	15,625	7.296e+00	4	201	0.9	7.296e+00	10	370	1.6
OBSTCLBM	5625	7.231e+00	3	88	0.1	7.231e+00	5	167	0.3
OBSTCLBM	10,000	7.272e+00	4	119	0.4	7.272e+00	5	299	0.8
OBSTCLBM	15,625	7.296e+00	4	164	0.8	7.296e+00	4	157	0.7
OBSTCLBU	5625	7.231e+00	5	211	0.4	7.231e+00	4	266	0.4
OBSTCLBU	10,000	7.272e+00	4	163	0.5	7.272e+00	7	293	0.7
OBSTCLBU	15,625	7.296e+00	5	231	1.1	7.296e+00	10	380	1.5
ODNAMUR	11,130	9.237e+03	722	40,213	24.5	9.237e+03	842	58,579	41.7
PENTDI	1000	−7.500e−01	3	11	0.0	−7.500e−01	3	14	0.0
PENTDI	5000	−7.500e−01	3	11	0.0	−7.500e−01	3	14	0.1
PENTDI	10,000	−7.500e−01	3	11	0.0	−7.500e−01	3	14	0.2
PENTDI	50,000	−7.500e−01	3	11	0.1	−7.500e−01	3	14	0.8
POWELLBC	1000	3.103e+05	483	4169	59.3	3.103e+05	287	3328	48.7
QRTQUAD	1200	−3.685e+09	118	638	0.1	−3.685e+09	201	660	0.1
QRTQUAD	5000	−2.649e+11	716	3078	1.1	−2.649e+11	1024	3908	1.8
QUDLIN	1200	−7.200e+07	4	1	0.0	−7.200e+07	4	2	0.0
QUDLIN	5000	−1.250e+09	7	2	0.0	−1.250e+09	7	4	0.0
QUDLIN	10,000	−5.000e+09	8	2	0.0	−5.000e+09	8	4	0.0
SCOND1LS	1002	2.967e−04	3657	944,441	57.3	8.954e−04	4850	2,576,566	184.4
SCOND1LS	5002	1.278e−02	1874	451,965	133.3	2.032e−02	1051	995,227	317.3
SINEALI	1000	−9.990e+04	16	44	0.0	−9.990e+04	14	83	0.0
TORSION1	1024	−4.450e−01	3	77	0.0	−4.450e−01	5	113	0.0
TORSION1	5476	−4.303e−01	4	213	0.3	−4.303e−01	15	333	0.4
TORSION1	10,000	−4.273e−01	4	265	0.6	−4.273e−01	20	556	1.3
TORSION1	14,884	−4.257e−01	5	338	1.2	−4.257e−01	27	753	2.4
TORSION2	1024	−4.450e−01	3	67	0.0	−4.450e−01	5	123	0.0
TORSION2	5476	−4.303e−01	4	151	0.3	−4.303e−01	10	334	0.5
TORSION2	10,000	−4.273e−01	4	264	0.8	−4.273e−01	12	466	1.1
TORSION2	14,884	−4.257e−01	4	260	1.1	−4.257e−01	15	616	2.0
TORSION3	1024	−1.232e+00	3	41	0.0	−1.232e+00	4	37	0.0
TORSION3	5476	−1.217e+00	3	84	0.1	−1.217e+00	6	127	0.1
TORSION3	10,000	−1.214e+00	4	143	0.3	−1.214e+00	7	205	0.3
TORSION3	14,884	−1.212e+00	4	156	0.4	−1.212e+00	9	245	0.6
TORSION4	1024	−1.232e+00	3	37	0.0	−1.232e+00	4	52	0.0
TORSION4	5476	−1.217e+00	3	102	0.1	−1.217e+00	6	165	0.2
TORSION4	10,000	−1.214e+00	4	131	0.3	−1.214e+00	7	249	0.5
TORSION4	14,884	−1.212e+00	5	221	0.8	−1.212e+00	10	308	0.9
TORSION5	1024	−2.876e+00	3	15	0.0	−2.876e+00	3	21	0.0
TORSION5	5476	−2.863e+00	3	39	0.0	−2.863e+00	4	53	0.0
TORSION5	10,000	−2.860e+00	3	62	0.1	−2.860e+00	4	79	0.1
TORSION5	14,884	−2.859e+00	3	68	0.2	−2.859e+00	5	92	0.2
TORSION6	1024	−2.876e+00	3	20	0.0	−2.876e+00	3	36	0.0
TORSION6	5476	−2.863e+00	3	47	0.0	−2.863e+00	4	91	0.1
TORSION6	10,000	−2.860e+00	3	65	0.1	−2.860e+00	4	130	0.3
TORSION6	14,884	−2.859e+00	4	116	0.3	−2.859e+00	5	173	0.5
TORSIONA	1024	−4.174e−01	3	78	0.0	−4.174e−01	5	115	0.0
TORSIONA	5476	−4.183e−01	4	222	0.3	−4.183e−01	15	330	0.4
TORSIONA	10,000	−4.184e−01	5	290	0.8	−4.184e−01	21	536	1.2
TORSIONA	5476	−4.183e−01	4	222	0.3	−4.183e−01	15	330	0.4
TORSIONB	1024	−4.174e−01	3	78	0.0	−4.174e−01	4	100	0.0
TORSIONB	5476	−4.183e−01	4	164	0.3	−4.183e−01	9	318	0.5
TORSIONB	10,000	−4.184e−01	5	261	0.8	−4.184e−01	10	467	1.2
TORSIONB	14,884	−4.184e−01	5	284	1.2	−4.184e−01	13	615	2.2
TORSIONC	1024	−1.202e+00	3	42	0.0	−1.202e+00	4	33	0.0
TORSIONC	5476	−1.204e+00	3	84	0.1	−1.204e+00	5	127	0.1
TORSIONC	10,000	−1.204e+00	4	139	0.3	−1.204e+00	7	205	0.3
TORSIONC	14,884	−1.204e+00	4	156	0.4	−1.204e+00	9	246	0.6
TORSIOND	1024	−1.202e+00	3	49	0.0	−1.202e+00	5	50	0.0
TORSIOND	5476	−1.204e+00	4	123	0.2	−1.204e+00	6	167	0.2
TORSIOND	10,000	−1.204e+00	4	142	0.3	−1.204e+00	9	234	0.5
TORSIOND	14,884	−1.204e+00	7	339	1.3	−1.204e+00	10	312	0.9
TORSIONE	1024	−2.846e+00	3	15	0.0	−2.846e+00	3	21	0.0
TORSIONE	5476	−2.850e+00	3	35	0.0	−2.850e+00	4	53	0.0
TORSIONE	10,000	−2.851e+00	3	56	0.1	−2.851e+00	4	79	0.1
TORSIONE	14,884	−2.851e+00	3	68	0.2	−2.851e+00	5	92	0.2
TORSIONF	1024	−2.846e+00	4	23	0.0	−2.846e+00	4	37	0.0
TORSIONF	5476	−2.850e+00	3	42	0.0	−2.850e+00	5	93	0.1
TORSIONF	10,000	−2.851e+00	3	66	0.1	−2.851e+00	4	131	0.3
TORSIONF	14,884	−2.851e+00	3	106	0.3	−2.851e+00	5	211	0.6

In Table 2, we report the 43 problems that have been solved in more than 1 s by at least one algorithm and both algorithms have found the same stationary point (with a tolerance of $10^{-3}$). To be more specific, let $f_{A}$ and $f_{N}$ be the objective function values at the stationary points found, respectively, by ASA-BCP  and NMBC  when applied to a particular problem. Let $f_{\min}=\min\{f_{A},f_{N}\}$. We consider that ASA-BCP  and NMBC  have found the same stationary point if

$$\frac{\left|f_{A}-f_{\min}\right|}{\max\{1,f_{\min}\}}<10^{-3}\;\mathrm{and}\;\frac{\left|f_{N}-f_{\min}\right|}{\max\{1,f_{\min}\}}<10^{-3}.$$

**Table 2:** Comparison between ASA-BCP  and NMBC  on the 43 problems that have been solved in more than 1 s by at least one algorithm and both algorithms have found the same stationary point (with a tolerance of $10^{-3}$).

**Problem**	n	**ASA-BCP**				**NMBC**			
		obj	f-eval	cg-it	time (s)	obj	f-eval	cg-it	time (s)
BIGGSB1	10,000	1.595e−02	199	2442	1.3	1.500e−02	6	6598	2.9
BIGGSB1	20,000	1.595e−02	199	2442	2.5	1.500e−02	7	19,025	16.6
BQPGAUSS	2003	−3.626e−01	9	9040	1.8	−3.626e−01	60	7674	1.6
CHARDIS0	10,000	1.246e−19	2	1	2.8	1.526e−16	2	2	3.3
CHENHARK	1000	−2.000e+00	107	1953	0.1	−2.000e+00	107	56,216	2.2
CHENHARK	5000	−2.000e+00	73	1556	0.4	−2.000e+00	89	129,087	23.0
CHENHARK	10,000	−2.000e+00	108	1935	0.9	−2.000e+00	117	278,775	105.7
CHENHARK	50,000	−2.000e+00	137	2643	4.5	−2.000e+00	61	207,468	348.6
JNLBRNG1	10,000	−1.806e−01	5	391	1.1	−1.806e−01	11	699	1.9
JNLBRNG1	15,625	−1.806e−01	5	429	1.6	−1.806e−01	13	955	3.7
JNLBRNG2	10,000	−4.149e+00	6	527	1.3	−4.149e+00	8	752	1.9
JNLBRNG2	15,625	−4.150e+00	7	659	2.3	−4.150e+00	10	969	3.4
JNLBRNGA	10,000	−2.711e−01	6	418	1.1	−2.711e−01	11	650	1.7
JNLBRNGA	15,625	−2.685e−01	5	446	1.8	−2.685e−01	13	907	3.4
JNLBRNGB	5625	−6.330e+00	6	1463	1.7	−6.330e+00	4	1388	1.7
JNLBRNGB	10,000	−6.301e+00	7	1962	3.7	−6.301e+00	4	2333	4.6
JNLBRNGB	15,625	−6.281e+00	8	2361	7.1	−6.281e+00	7	2902	8.9
MCCORMCK	10,000	−9.133e+03	12	16	0.1	−9.133e+03	38	547	1.1
MCCORMCK	50,000	−4.566e+04	18	29	0.5	−4.566e+04	37	255	2.5
MINSURFO	2706	2.515e+00	5	274	0.3	2.515e+00	26	2390	2.6
MINSURFO	5931	2.485e+00	105	647	1.8	2.485e+00	27	1994	4.2
NCVXBQP3	10,000	−6.506e+09	54	129	0.1	−6.506e+09	6164	15,326	9.8
NCVXBQP3	100,000	−6.505e+11	287	1024	6.6	−6.505e+11	679	3076	16.7
NOBNDTOR	10,000	−4.438e−01	5	318	0.9	−4.438e−01	16	763	2.3
NOBNDTOR	14,884	−4.405e−01	6	396	1.7	−4.405e−01	19	914	3.8
NOBNDTOR	40,000	−4.347e−01	9	659	7.6	−4.347e−01	34	1981	20.9
OBSTCLAE	10,000	1.886e+00	23	438	1.6	1.886e+00	5	384	0.9
OBSTCLAE	15,625	1.901e+00	16	406	1.9	1.901e+00	9	541	1.8
OBSTCLAL	15,625	1.901e+00	4	211	0.7	1.901e+00	8	433	1.2
OBSTCLBL	15,625	7.296e+00	4	201	0.9	7.296e+00	10	370	1.6
OBSTCLBU	15,625	7.296e+00	5	231	1.1	7.296e+00	10	380	1.5
ODNAMUR	11,130	9.237e+03	722	40,213	24.5	9.237e+03	842	58,579	41.7
POWELLBC	1000	3.103e+05	483	4169	59.3	3.103e+05	287	3328	48.7
QRTQUAD	5000	−2.649e+11	716	3078	1.1	−2.649e+11	1024	3908	1.8
SCOND1LS	1002	2.967e−04	3657	944,441	57.3	8.954e−04	4850	2,576,566	184.4
TORSION1	10,000	−4.273e−01	4	265	0.6	−4.273e−01	20	556	1.3
TORSION1	14,884	−4.257e−01	5	338	1.2	−4.257e−01	27	753	2.4
TORSION2	10,000	−4.273e−01	4	264	0.8	−4.273e−01	12	466	1.1
TORSION2	14,884	−4.257e−01	4	260	1.1	−4.257e−01	15	616	2.0
TORSIONA	10,000	−4.184e−01	5	290	0.8	−4.184e−01	21	536	1.2
TORSIONB	10,000	−4.184e−01	5	261	0.8	−4.184e−01	10	467	1.2
TORSIONB	14,884	−4.184e−01	5	284	1.2	−4.184e−01	13	615	2.2
TORSIOND	14,884	−1.204e+00	7	339	1.3	−1.204e+00	10	312	0.9

In Table 3, we report the problems that have been solved in more than 1 s by ASA-BCP  or NMBC  and such that both algorithms have found different stationary points (with a tolerance of $10^{-3}$).

**Table 3:** Comparison between ASA-BCP and NMBC: problems solved in more than 1 s by at least one algorithm and such that both algorithms find different stationary points.

**Problem**	n	**ASA-BCP**				**NMBC**			
		obj	f-eval	cg-it	time (s)	obj	f-eval	cg-it	time (s)
SCOND1LS	5002	1.278e−02	1874	451,965	133.3	2.032e−02	1051	995,227	317.3

All the tables include the following data: the name (Problem) and the dimension (*n*) of the problems considered, the objective function value at the stationary point found (obj), the number of function evaluations (f-eval), the total number of conjugate gradient iterations (cg-it) and the computational time in seconds (time).

### 2.5 Comparison among ASA-BCP, ALGENCAN  and LANCELOT B

In Table 4, we report the numerical results obtained by ASA-BCP, ALGENCAN  and LANCELOT B  on 140 problems from the CUTEst collection.

**Table 4:** Comparison among ASA-BCP, ALGENCAN and LANCELOT B on 140 problems from the CUTEst collection.

**Problem**	n	**ASA-BCP**				**ALGENCAN**				**LANCELOT B**			
		obj	f-eval	cg-it	time (s)	obj	f-eval	cg-it	time (s)	obj	f-eval	cg-it	time (s)
BDEXP	1000	3.953e−04	11	10	0.0	3.919e−04	11	69	0.0	3.919e−04	11	19	0.0
BDEXP	5000	1.967e−03	11	10	0.0	1.964e−03	11	59	0.0	1.964e−03	11	26	0.1
BIGGSB1	1000	1.500e−02	198	2272	0.1	1.500e−02	4034	$>10^{5}$	2.5	1.500e−02	502	500	0.1
BIGGSB1	5000	1.595e−02	199	2442	0.6	1.500e−02	26,150	$>10^{6}$	283.3	1.500e−02	2502	2500	2.1
BIGGSB1	10,000	1.595e−02	199	2442	1.3	1.500e−02	57,308	$>10^{7}$	2274.7	1.500e−02	5002	5000	7.4
BIGGSB1	20,000	1.595e−02	199	2442	2.5	1.500e−02	$>10^{5}$	$>10^{7}$	18,287.8	1.500e−02	10,002	10,000	32.6
BQPGAUSS	2003	−3.626e−01	9	9040	1.8	−3.626e−01	189	20,144	1.3	−3.626e−01	12	4903	1.1
CHARDIS0	2000	6.384e−22	2	1	0.1	1.618e−21	2	2	0.3	7.275e−22	2	0	7.2
CHARDIS0	4000	4.648e−20	2	1	0.5	1.668e−20	2	2	1.1	5.438e−20	2	0	76.7
CHARDIS0	10,000	1.246e−19	2	1	2.8	1.793e−19	2	2	6.6	1.799e−18	2	0	2226.3
CHENHARK	1000	−2.000e+00	107	1953	0.1	−2.000e+00	64	4670	0.1	−2.000e+00	206	484	0.1
CHENHARK	5000	−2.000e+00	73	1556	0.4	−2.000e+00	36	6091	0.6	−2.000e+00	611	1641	0.9
CHENHARK	10,000	−2.000e+00	108	1935	0.9	−2.000e+00	35	6092	1.3	−2.000e+00	615	2294	2.0
CHENHARK	50,000	−2.000e+00	137	2643	4.5	−2.00e+00	35	10,094	7.2	−2.000e+00	589	3953	7.9
CVXBQP1	1000	2.252e+04	10	9	0.0	2.252e+04	11	5	0.0	2.252e+04	2	0	0.0
CVXBQP1	10,000	2.250e+06	13	11	0.0	2.250e+06	14	5	0.0	2.250e+06	2	1	0.1
CVXBQP1	100,000	2.250e+08	15	12	0.2	2.250e+08	18	5	0.1	2.250e+08	2	0	16.0
EXPLIN	1200	−7.193e+07	108	279	0.0	−7.192e+07	91	292	0.0	−7.193e+07	14	285	0.0
EXPLIN2	1200	−7.200e+07	41	69	0.0	−7.200e+07	109	129	0.0	−7.200e+07	10	70	0.0
EXPQUAD	1200	−3.685e+09	146	225	0.0	−3.685e+09	185	484	0.0	−3.685e+09	100	373	0.1
JNLBRNG1	1024	−1.803e−01	4	135	0.0	−1.803e−01	28	374	0.0	−1.803e−01	7	93	0.0
JNLBRNG1	1156	−1.803e−01	3	128	0.0	−1.803e−01	35	439	0.0	−1.803e−01	8	117	0.0
JNLBRNG1	5625	−1.805e−01	4	278	0.4	−1.805e−01	92	1788	0.3	−1.805e−01	15	635	1.0
JNLBRNG1	10,000	−1.806e−01	5	391	1.1	−1.806e−01	121	3239	0.9	−1.806e−01	20	1135	2.9
JNLBRNG1	15,625	−1.806e−01	5	429	1.6	−1.806e−01	163	4889	2.0	−1.806e−01	25	1810	7.1
JNLBRNG2	1024	−4.125e+00	3	158	0.0	−4.125e+00	12	342	0.0	−4.125e+00	5	68	0.0
JNLBRNG2	1156	−4.128e+00	3	191	0.1	−4.128e+00	13	362	0.0	−4.128e+00	5	68	0.0
JNLBRNG2	5625	−4.147e+00	5	407	0.5	−4.147e+00	44	2155	0.3	−4.147e+00	10	319	0.5
JNLBRNG2	10,000	−4.149e+00	6	527	1.3	−4.149e+00	59	3376	0.8	−4.149e+00	12	537	1.3
JNLBRNG2	15,625	−4.150e+00	7	659	2.3	−4.150e+00	79	5154	1.9	−4.150e+00	15	912	3.4
JNLBRNGA	1024	−2.954e−01	3	99	0.0	−2.954e−01	28	253	0.0	−2.954e−01	8	81	0.0
JNLBRNGA	1156	−2.935e−01	3	120	0.0	−2.935e−01	29	253	0.0	−2.935e−01	8	85	0.0
JNLBRNGA	5625	−2.753e−01	5	340	0.5	−2.753e−01	75	734	0.2	−2.753e−01	15	441	0.7
JNLBRNGA	10000	−2.711e−01	6	418	1.1	−2.711e−01	106	1199	0.5	−2.711e−01	19	824	2.2
JNLBRNGA	15625	−2.685e−01	5	446	1.8	−2.685e−01	129	1508	0.8	−2.685e−01	22	1327	5.2
JNLBRNGB	1024	−6.440e+00	4	596	0.1	−6.440e+00	11	841	0.0	−6.440e+00	8	48	0.0
JNLBRNGB	1156	−6.429e+00	4	593	0.1	−6.429e+00	17	1137	0.0	−6.429e+00	8	52	0.0
JNLBRNGB	5625	−6.330e+00	6	1463	1.7	−6.330e+00	26	2744	0.4	−6.330e+00	10	106	0.2
JNLBRNGB	10,000	−6.301e+00	7	1962	3.7	−6.301e+00	51	3418	0.9	−6.301e+00	10	156	0.5
JNLBRNGB	15,625	−6.281e+00	8	2361	7.1	−6.281e+00	56	5074	1.9	−6.281e+00	11	329	1.3
LINVERSE	1999	6.810e+02	21	241	0.1	6.820e+02	25	71	0.0	6.810e+02	23	1645	0.5
MCCORMCK	1000	−9.137e+02	7	20	0.0	−9.137e+02	11	27	0.0	−9.137e+02	8	4	0.0
MCCORMCK	5000	−4.567e+03	13	22	0.0	−4.567e+03	10	29	0.0	−4.567e+03	7	5	0.0
MCCORMCK	10,000	−9.133e+03	12	16	0.1	−9.133e+03	10	29	0.1	−9.133e+03	7	4	0.1
MCCORMCK	50,000	−4.566e+04	18	29	0.5	−4.566e+04	10	29	0.3	−4.566e+04	8	6	0.3
MINSURFO	2706	2.515e+00	5	274	0.3	2.515e+00	8	525	0.1	2.515e+00	7	53	0.1
MINSURFO	5931	2.485e+00	105	647	1.8	2.485e+00	10	948	0.2	2.485e+00	9	119	0.3
NCVXBQP1	1000	−1.987e+08	10	9	0.0	−1.987e+08	4	1	0.0	−1.987e+08	2	0	0.0
NCVXBQP1	10,000	−1.986e+10	11	10	0.0	−1.986e+10	8	1	0.0	−1.986e+10	2	0	0.1
NCVXBQP1	100,000	−1.985e+12	15	11	0.2	−1.985e+12	11	1	0.1	−1.985e+12	2	6	16.1
NCVXBQP2	1000	−1.334e+08	15	24	0.0	−1.334e+08	26	24	0.0	−1.334e+08	3	39	0.0
NCVXBQP2	10,000	−1.334e+10	31	63	0.1	−1.334e+10	84	102	0.1	−1.334e+10	4	407	0.2
NCVXBQP2	100,000	−1.334e+12	41	100	0.7	−1.334e+12	87	142	1.2	−1.334e+12	4	4013	18.5
NCVXBQP3	1000	−6.530e+07	20	34	0.0	−6.577e+07	13	18	0.0	−6.579e+07	4	34	0.0
NCVXBQP3	10,000	−6.506e+09	54	129	0.1	−6.557e+09	29	50	0.0	−6.558e+09	6	360	0.2
NCVXBQP3	100,000	−6.505e+11	287	1024	6.6	−6.556e+11	69	185	1.1	−6.557e+11	6	3441	20.3
NOBNDTOR	5476	−4.499e−01	4	235	0.4	−4.499e−01	126	1725	0.4	−4.499e−01	24	363	0.7
NOBNDTOR	10,000	−4.438e−01	5	318	0.9	−4.438e−01	173	2630	0.9	−4.438e−01	31	596	2.0
NOBNDTOR	14,884	−4.405e−01	6	396	1.7	−4.405e−01	224	3763	1.9	−4.405e−01	37	790	4.0
NOBNDTOR	40,000	−4.347e−01	9	659	7.6	−4.347e−01	408	8922	11.7	−4.347e−01	61	1791	23.9
NONSCOMP	1000	8.661e−16	7	37	0.0	2.981e−15	9	62	0.0	2.986e−15	9	8	0.0
NONSCOMP	5000	5.054e−13	7	34	0.0	1.529e−14	9	59	0.0	1.523e−14	9	9	0.0
NONSCOMP	10,000	1.680e−12	7	37	0.0	3.062e−14	9	56	0.0	3.055e−14	9	9	0.0
OBSTCLAE	5625	1.863e+00	9	221	0.4	1.863e+00	50	788	0.2	1.863e+00	6	2488	4.7
OBSTCLAE	10,000	1.886e+00	23	438	1.6	1.886e+00	85	945	0.6	1.886e+00	7	4646	15.1
OBSTCLAE	15,625	1.901e+00	16	406	1.9	1.901e+00	105	2244	1.0	1.901e+00	5	7409	37.5
OBSTCLAL	5625	1.863e+00	4	124	0.2	1.863e+00	63	789	0.2	1.863e+00	17	196	0.3
OBSTCLAL	10,000	1.886e+00	4	159	0.3	1.886e+00	83	1224	0.4	1.886e+00	20	336	0.7
OBSTCLAL	15,625	1.901e+00	4	211	0.7	1.901e+00	112	1862	0.9	1.901e+00	25	480	1.6
OBSTCLBL	5625	7.231e+00	4	116	0.2	7.231e+00	51	323	0.2	7.231e+00	13	953	1.5
OBSTCLBL	10,000	7.272e+00	4	155	0.5	7.272e+00	94	435	0.5	7.272e+00	16	1797	4.9
OBSTCLBL	15,625	7.296e+00	4	201	0.9	7.296e+00	110	516	1.1	7.296e+00	19	2761	11.8
OBSTCLBM	5625	7.231e+00	3	88	0.1	7.231e+00	20	181	0.1	7.231e+00	6	485	0.9
OBSTCLBM	10,000	7.272e+00	4	119	0.4	7.272e+00	32	285	0.2	7.272e+00	6	794	2.6
OBSTCLBM	15,625	7.296e+00	4	164	0.8	7.296e+00	34	310	0.3	7.296e+00	6	1377	7.1
OBSTCLBU	5625	7.231e+00	5	211	0.4	7.231e+00	43	371	0.1	7.231e+00	14	230	0.4
OBSTCLBU	10,000	7.272e+00	4	163	0.5	7.272e+00	60	478	0.3	7.272e+00	17	425	1.2
OBSTCLBU	15,625	7.296e+00	5	231	1.1	7.296e+00	81	687	0.7	7.296e+00	20	787	3.3
ODNAMUR	11,130	9.237e+03	722	40,213	24.5	9.237e+03	368	$>10^{5}$	3037.8	9.237e+03	12	27,886	23.6
PENTDI	1000	−7.500e−01	3	11	0.0	−7.500e−01	2	0	0.0	−7.500e−01	2	0	0.0
PENTDI	5000	−7.500e−01	3	11	0.0	−7.500e−01	2	0	0.0	−7.500e−01	2	0	0.0
PENTDI	10,000	−7.500e−01	3	11	0.0	−7.500e−01	2	0	0.0	−7.500e−01	2	0	0.0
PENTDI	50,000	−7.500e−01	3	11	0.1	−7.500e−01	2	0	0.0	−7.500e−01	2	0	0.0
POWELLBC	1000	3.103e+05	483	4169	59.3	3.103e+05	361	2971	17.7	3.198e+05	635	3296	79.7
QRTQUAD	1200	−3.685e+09	118	638	0.1	−3.685e+09	206	851	0.0	−3.685e+09	230	435	0.3
QRTQUAD	5000	−2.649e+11	716	3078	1.1	−2.649e+11	531	2822	0.5	−2.649e+11	3568	11,356	34.0
QUDLIN	1200	−7.200e+07	4	1	0.0	−7.200e+07	2	0	0.0	−7.200e+07	2	0	0.0
QUDLIN	5000	−1.250e+09	7	2	0.0	−1.250e+09	2	0	0.0	−1.250e+09	2	0	0.0
QUDLIN	10,000	−5.000e+09	8	2	0.0	−5.000e+09	2	0	0.0	−5.000e+09	2	0	0.1
SCOND1LS	1002	2.967e−04	3657	$>10^{5}$	57.3	8.938e−06	1045	$>10^{5}$	12.3	1.158e−10	645	618	0.3
SCOND1LS	5002	1.278e−02	1874	$>10^{5}$	133.3	2.391e−04	1060	$>10^{6}$	318.7	3.418e−05	740	716	1.8
SINEALI	1000	−9.990e+04	16	44	0.0	−9.990e+04	31	64	0.0	−9.990e+04	14	10	0.0
TORSION1	1024	−4.450e−01	3	77	0.0	−4.450e−01	33	172	0.0	−4.450e−01	11	73	0.0
TORSION1	5476	−4.303e−01	4	213	0.3	−4.303e−01	116	1027	0.3	−4.303e−01	24	321	0.5
TORSION1	10,000	−4.273e−01	4	265	0.6	−4.273e−01	173	1791	0.7	−4.273e−01	32	549	1.5
TORSION1	14,884	−4.257e−01	5	338	1.2	−4.257e−01	217	2498	1.3	−4.257e−01	38	793	3.1
TORSION2	1024	−4.450e−01	3	67	0.0	−4.450e−01	30	129	0.0	−4.450e−01	7	301	0.1
TORSION2	5476	−4.303e−01	4	151	0.3	−4.303e−01	87	562	0.2	−4.303e−01	10	1640	3.1
TORSION2	10,000	−4.273e−01	4	264	0.8	−4.273e−01	117	817	0.4	−4.273e−01	10	3138	11.0
TORSION2	14,884	−4.257e−01	4	260	1.1	−4.257e−01	143	983	0.7	−4.257e−01	10	4326	22.6
TORSION3	1024	−1.232e+00	3	41	0.0	−1.232e+00	12	45	0.0	−1.232e+00	6	23	0.0
TORSION3	5476	−1.217e+00	3	84	0.1	−1.217e+00	43	292	0.1	−1.217e+00	12	98	0.1
TORSION3	10,000	−1.214e+00	4	143	0.3	−1.214e+00	66	525	0.2	−1.214e+00	16	171	0.4
TORSION3	14,884	−1.212e+00	4	156	0.4	−1.212e+00	92	807	0.5	−1.212e+00	20	241	0.7
TORSION4	1024	−1.232e+00	3	37	0.0	−1.232e+00	18	71	0.0	−1.232e+00	7	256	0.1
TORSION4	5476	−1.217e+00	3	102	0.1	−1.217e+00	50	201	0.1	−1.217e+00	10	1387	2.3
TORSION4	10000	−1.214e+00	4	131	0.3	−1.214e+00	72	319	0.2	−1.214e+00	12	4572	14.0
TORSION4	14884	−1.212e+00	5	221	0.8	−1.212e+00	102	464	0.4	−1.212e+00	16	5646	27.5
TORSION5	1024	−2.876e+00	3	15	0.0	−2.876e+00	6	21	0.0	−2.876e+00	4	7	0.0
TORSION5	5476	−2.863e+00	3	39	0.0	−2.863e+00	18	103	0.0	−2.863e+00	7	33	0.0
TORSION5	10,000	−2.860e+00	3	62	0.1	−2.860e+00	27	171	0.1	−2.860e+00	9	57	0.1
TORSION5	14,884	−2.859e+00	3	68	0.2	−2.859e+00	33	215	0.2	−2.859e+00	10	72	0.2
TORSION6	1024	−2.876e+00	3	20	0.0	−2.876e+00	11	24	0.0	−2.876e+00	6	171	0.0
TORSION6	5476	−2.863e+00	3	47	0.0	−2.863e+00	26	76	0.1	−2.863e+00	8	2002	2.2
TORSION6	10,000	−2.860e+00	3	65	0.1	−2.860e+00	37	110	0.1	−2.860e+00	9	4400	8.5
TORSION6	14,884	−2.859e+00	4	116	0.3	−2.859e+00	43	111	0.2	−2.859e+00	11	4933	16.8
TORSIONA	1024	−4.174e−01	3	78	0.0	−4.174e−01	33	172	0.0	−4.174e−01	11	77	0.0
TORSIONA	5476	−4.183e−01	4	222	0.3	−4.183e−01	116	1034	0.3	−4.183e−01	24	320	0.6
TORSIONA	10,000	−4.184e−01	5	290	0.8	−4.184e−01	173	1802	0.7	−4.184e−01	32	558	1.6
TORSIONA	5476	−4.183e−01	4	222	0.3	−4.183e−01	116	1034	0.3	−4.183e−01	24	320	0.5
TORSIONB	1024	−4.174e−01	3	78	0.0	−4.174e−01	25	126	0.0	−4.174e−01	8	149	0.1
TORSIONB	5476	−4.183e−01	4	164	0.3	−4.183e−01	73	488	0.1	−4.183e−01	8	1677	3.3
TORSIONB	10,000	−4.184e−01	5	261	0.8	−4.184e−01	101	714	0.4	−4.184e−01	10	2593	9.5
TORSIONB	14,884	−4.184e−01	5	284	1.2	−4.184e−01	127	909	0.7	−4.184e−01	10	4447	24.0
TORSIONC	1024	−1.202e+00	3	42	0.0	−1.202e+00	12	44	0.0	−1.202e+00	6	23	0.0
TORSIONC	5476	−1.204e+00	3	84	0.1	−1.204e+00	43	291	0.1	−1.204e+00	12	98	0.1
TORSIONC	10,000	−1.204e+00	4	139	0.3	−1.204e+00	66	526	0.3	−1.204e+00	16	171	0.4
TORSIONC	14,884	−1.204e+00	4	156	0.4	−1.204e+00	92	806	0.5	−1.204e+00	20	241	0.7
TORSIOND	1024	−1.202e+00	3	49	0.0	−1.202e+00	19	72	0.0	−1.202e+00	8	266	0.1
TORSIOND	5476	−1.204e+00	4	123	0.2	−1.204e+00	51	203	0.1	−1.204e+00	11	1399	1.9
TORSIOND	10,000	−1.204e+00	4	142	0.3	−1.204e+00	71	322	0.3	−1.204e+00	16	2136	7.0
TORSIOND	14,884	−1.204e+00	7	339	1.3	−1.204e+00	103	468	0.5	−1.204e+00	10	9134	41.2
TORSIONE	1024	−2.846e+00	3	15	0.0	−2.846e+00	6	20	0.0	−2.846e+00	4	7	0.0
TORSIONE	5476	−2.850e+00	3	35	0.0	−2.850e+00	18	102	0.1	−2.850e+00	7	33	0.0
TORSIONE	10,000	−2.851e+00	3	56	0.1	−2.851e+00	27	170	0.1	−2.851e+00	9	57	0.1
TORSIONE	14,884	−2.851e+00	3	68	0.2	−2.851e+00	33	215	0.2	−2.851e+00	10	72	0.2
TORSIONF	1024	−2.846e+00	4	23	0.0	−2.846e+00	11	41	0.0	−2.846e+00	6	190	0.0
TORSIONF	5476	−2.850e+00	3	42	0.0	−2.850e+00	26	124	0.1	−2.850e+00	8	1960	2.2
TORSIONF	10,000	−2.851e+00	3	66	0.1	−2.851e+00	33	184	0.2	−2.851e+00	9	4416	8.9
TORSIONF	14,884	−2.851e+00	3	106	0.3	−2.851e+00	42	206	0.2	−2.851e+00	11	5112	18.3

In Table 5, we report the 62 problems that have been solved in more than 1 s by at least one algorithm and all the algorithms have found the same stationary point (with a tolerance of $10^{-3}$). To be more specific, let $f_{\mathrm{AS}}$, $f_{\mathrm{AL}}$ and $f_{\mathrm{LB}}$ be the objective function values at the stationary points found, respectively, by ASA-BCP, ALGENCAN  and LANCELOT B  when applied to a particular problem. Let $f_{\min}=\min\{f_{\mathrm{AS}},f_{\mathrm{AL}},f_{\mathrm{LB}}\}$. We consider that ASA-BCP, ALGENCAN  and LANCELOT B  find the same stationary point if

$$\frac{\left|f_{\mathrm{AS}}-f_{\min}\right|}{\max\{1,f_{\min}\}}<10^{-3},\;\frac{\left|f_{\mathrm{AL}}-f_{\min}\right|}{\max\{1,f_{\min}\}}<10^{-3}\;\mathrm{and}\;\frac{\left|f_{\mathrm{LB}}-f_{\min}\right|}{\max\{1,f_{\min}\}}<10^{-3}.$$

**Table 5:** Comparison between ASA-BCP, ALGENCAN and LANCELOT B on the 62 problems that have been solved in more than 1 s by at least one algorithm and all the algorithms have found the same stationary point (with a tolerance of $10^{-3}$).

**Problem**	n	**ASA-BCP**				**ALGENCAN**				**LANCELOT B**			
		obj	f-eval	cg-it	time (s)	obj	f-eval	cg-it	time (s)	obj	f-eval	cg-it	time (s)
BIGGSB1	1000	1.500e−02	198	2272	0.1	1.500e−02	4034	$>10^{5}$	2.5	1.500e−02	502	500	0.1
BIGGSB1	5000	1.595e−02	199	2442	0.6	1.500e−02	26,150	$>10^{6}$	283.3	1.500e−02	2502	2500	2.1
BIGGSB1	10,000	1.595e−02	199	2442	1.3	1.500e−02	57,308	$>10^{7}$	2274.7	1.500e−02	5002	5000	7.4
BIGGSB1	20,000	1.595e−02	199	2442	2.5	1.500e−02	$>10^{5}$	$>10^{7}$	18,287.8	1.500e−02	10,002	10,000	32.6
BQPGAUSS	2003	−3.626e−01	9	9040	1.8	−3.626e−01	189	20,144	1.3	−3.626e−01	12	4903	1.1
CHARDIS0	2000	6.384e−22	2	1	0.1	1.618e−21	2	2	0.3	7.275e−22	2	0	7.2
CHARDIS0	4000	4.648e−20	2	1	0.5	1.668e−20	2	2	1.1	5.438e−20	2	0	76.7
CHARDIS0	10,000	1.246e−19	2	1	2.8	1.793e−19	2	2	6.6	1.799e−18	2	0	2226.3
CHENHARK	10,000	−2.000e+00	108	1935	0.9	−2.000e+00	35	6092	1.3	−2.000e+00	615	2294	2.0
CHENHARK	50,000	−2.000e+00	137	2643	4.5	−2.000e+00	35	10,094	7.2	−2.000e+00	589	3953	7.9
CVXBQP1	100,000	2.250e+08	15	12	0.2	2.250e+08	18	5	0.1	2.250e+08	2	0	16.0
JNLBRNG1	5625	−1.805e−01	4	278	0.4	−1.805e−01	92	1788	0.3	−1.805e−01	15	635	1.0
JNLBRNG1	10,000	−1.806e−01	5	391	1.1	−1.806e−01	121	3239	0.9	−1.806e−01	20	1135	2.9
JNLBRNG1	15,625	−1.806e−01	5	429	1.6	−1.806e−01	163	4889	2.0	−1.806e−01	25	1810	7.1
JNLBRNG2	10,000	−4.149e+00	6	527	1.3	−4.149e+00	59	3376	0.8	−4.149e+00	12	537	1.3
JNLBRNG2	15,625	−4.150e+00	7	659	2.3	−4.150e+00	79	5154	1.9	−4.150e+00	15	912	3.4
JNLBRNGA	10,000	−2.711e−01	6	418	1.1	−2.711e−01	106	1199	0.5	−2.711e−01	19	824	2.2
JNLBRNGA	15,625	−2.685e−01	5	446	1.8	−2.685e−01	129	1508	0.8	−2.685e−01	22	1327	5.2
JNLBRNGB	5625	−6.330e+00	6	1463	1.7	−6.330e+00	26	2744	0.4	−6.330e+00	10	106	0.2
JNLBRNGB	10,000	−6.301e+00	7	1962	3.7	−6.301e+00	51	3418	0.9	−6.301e+00	10	156	0.5
JNLBRNGB	15,625	−6.281e+00	8	2361	7.1	−6.281e+00	56	5074	1.9	−6.281e+00	11	329	1.3
MINSURFO	5931	2.485e+00	105	647	1.8	2.485e+00	10	948	0.2	2.485e+00	9	119	0.3
NCVXBQP1	100,000	−1.985e+12	15	11	0.2	−1.985e+12	11	1	0.1	−1.985e+12	2	6	16.1
NCVXBQP2	100,000	−1.334e+12	41	100	0.7	−1.334e+12	87	142	1.2	−1.334e+12	4	4013	18.5
NOBNDTOR	10,000	−4.438e−01	5	318	0.9	−4.438e−01	173	2630	0.9	−4.438e−01	31	596	2.0
NOBNDTOR	14,884	−4.405e−01	6	396	1.7	−4.405e−01	224	3763	1.9	−4.405e−01	37	790	4.0
NOBNDTOR	40,000	−4.347e−01	9	659	7.6	−4.347e−01	408	8922	11.7	−4.347e−01	61	1791	23.9
OBSTCLAE	5625	1.863e+00	9	221	0.4	1.863e+00	50	788	0.2	1.863e+00	6	2488	4.7
OBSTCLAE	10,000	1.886e+00	23	438	1.6	1.886e+00	85	945	0.6	1.886e+00	7	4646	15.1
OBSTCLAE	15,625	1.901e+00	16	406	1.9	1.901e+00	105	2244	1.0	1.901e+00	5	7409	37.5
OBSTCLAL	15,625	1.901e+00	4	211	0.7	1.901e+00	112	1862	0.9	1.901e+00	25	480	1.6
OBSTCLBL	5625	7.231e+00	4	116	0.2	7.231e+00	51	323	0.2	7.231e+00	13	953	1.5
OBSTCLBL	10,000	7.272e+00	4	155	0.5	7.272e+00	94	435	0.5	7.272e+00	16	1797	4.9
OBSTCLBL	15,625	7.296e+00	4	201	0.9	7.296e+00	110	516	1.1	7.296e+00	19	2761	11.8
OBSTCLBM	10,000	7.272e+00	4	119	0.4	7.272e+00	32	285	0.2	7.272e+00	6	794	2.6
OBSTCLBM	15,625	7.296e+00	4	164	0.8	7.296e+00	34	310	0.3	7.296e+00	6	1377	7.1
OBSTCLBU	10,000	7.272e+00	4	163	0.5	7.272e+00	60	478	0.3	7.272e+00	17	425	1.2
OBSTCLBU	15,625	7.296e+00	5	231	1.1	7.296e+00	81	687	0.7	7.296e+00	20	787	3.3
ODNAMUR	11,130	9.237e+03	722	40,213	24.5	9.237e+03	368	$>10^{5}$	3037.8	9.237e+03	12	27,886	23.6
QRTQUAD	5000	−2.649e+11	716	3078	1.1	−2.649e+11	531	2822	0.5	−2.649e+11	3568	11,356	34.0
SCOND1LS	1002	2.967e−04	3657	$>10^{5}$	57.3	8.938e−06	1045	$>10^{5}$	12.3	1.158e−10	645	618	0.3
TORSION1	10,000	−4.273e−01	4	265	0.6	−4.273e−01	173	1791	0.7	−4.273e−01	32	549	1.5
TORSION1	14,884	−4.257e−01	5	338	1.2	−4.257e−01	217	2498	1.3	−4.257e−01	38	793	3.1
TORSION2	5476	−4.303e−01	4	151	0.3	−4.303e−01	87	562	0.2	−4.303e−01	10	1640	3.1
TORSION2	10,000	−4.273e−01	4	264	0.8	−4.273e−01	117	817	0.4	−4.273e−01	10	3138	11.0
TORSION2	14,884	−4.257e−01	4	260	1.1	−4.257e−01	143	983	0.7	−4.257e−01	10	4326	22.6
TORSION4	5476	−1.217e+00	3	102	0.1	−1.217e+00	50	201	0.1	−1.217e+00	10	1387	2.3
TORSION4	10,000	−1.214e+00	4	131	0.3	−1.214e+00	72	319	0.2	−1.214e+00	12	4572	14.0
TORSION4	14,884	−1.212e+00	5	221	0.8	−1.212e+00	102	464	0.4	−1.212e+00	16	5646	27.5
TORSION6	5476	−2.863e+00	3	47	0.0	−2.863e+00	26	76	0.1	−2.863e+00	8	2002	2.2
TORSION6	10,000	−2.860e+00	3	65	0.1	−2.860e+00	37	110	0.1	−2.860e+00	9	4400	8.5
TORSION6	14,884	−2.859e+00	4	116	0.3	−2.859e+00	43	111	0.2	−2.859e+00	11	4933	16.8
TORSIONA	10,000	−4.184e−01	5	290	0.8	−4.184e−01	173	1802	0.7	−4.184e−01	32	558	1.6
TORSIONB	5476	−4.183e−01	4	164	0.3	−4.183e−01	73	488	0.1	−4.183e−01	8	1677	3.3
TORSIONB	10,000	−4.184e−01	5	261	0.8	−4.184e−01	101	714	0.4	−4.184e−01	10	2593	9.5
TORSIONB	14,884	−4.184e−01	5	284	1.2	−4.184e−01	127	909	0.7	−4.184e−01	10	4447	24.0
TORSIOND	5476	−1.204e+00	4	123	0.2	−1.204e+00	51	203	0.1	−1.204e+00	11	1399	1.9
TORSIOND	10,000	−1.204e+00	4	142	0.3	−1.204e+00	71	322	0.3	−1.204e+00	16	2136	7.0
TORSIOND	14,884	−1.204e+00	7	339	1.3	−1.204e+00	103	468	0.5	−1.204e+00	10	9134	41.2
TORSIONF	5476	−2.850e+00	3	42	0.0	−2.850e+00	26	124	0.1	−2.850e+00	8	1960	2.2
TORSIONF	10,000	−2.851e+00	3	66	0.1	−2.851e+00	33	184	0.2	−2.851e+00	9	4416	8.9
TORSIONF	14,884	−2.851e+00	3	106	0.3	−2.851e+00	42	206	0.2	−2.851e+00	11	5112	18.3

In Table 6, we report the problems that have been solved in more than 1 s by at least one algorithm among ASA-BCP, ALGENCAN  and LANCELOT B  and such that all the algorithms have found different stationary points (with a tolerance of $10^{-3}$).

**Table 6:** Comparison between ASA-BCP, ALGENCAN  and LANCELOT B: problems solved in more than 1 s by at least one algorithm and such that all the algorithms find different stationary points.

**Problem**	n	**ASA-BCP**				**ALGENCAN**				**LANCELOT B**			
		obj	f-eval	cg-it	time (s)	obj	f-eval	cg-it	time (s)	obj	f-eval	cg-it	time (s)
NCVXBQP3	100,000	−6.505e+11	287	1024	6.6	−6.556e+11	69	185	1.1	−6.557e+11	6	3441	20.3
POWELLBC	1000	3.103e+05	483	4169	59.3	3.103e+05	361	2971	17.7	3.198e+05	635	3296	79.7
SCOND1LS	5002	1.278e−02	1874	$>10^{5}$	133.3	2.391e−04	1060	$>10^{6}$	318.7	3.418e−05	740	716	1.8

All the tables include the following data: the name (Problem) and the dimension (*n*) of the problems considered, the objective function value at the stationary point found (obj), the number of function evaluations (f-eval), the total number of conjugate gradient iterations (cg-it) and the computational time in seconds (time).

## Appendix A Supporting Information

Application 1
