# Supplementary material for: Data and performance of an active-set truncated Newton method with non-monotone line search for bound-constrained optimization
Source: Data Brief. 2018 Nov 20;21:2155–69. doi: 10.1016/j.dib.2018.11.061 (PMC6265501; doi:10.1016/j.dib.2018.11.061)
Supplement: Application 1 [file mmc2.docx]

Supplementary material Supplementary data associated

with this article can be found in the online version at <https://doi.org/10.1007/s10957-016-1024-9>
